# Using techno-economic modelling to determine the minimum cost possible for a microbial palm oil substitute

**DOI:** 10.1186/s13068-021-01911-3

**Published:** 2021-03-04

**Authors:** Eleni E. Karamerou, Sophie Parsons, Marcelle C. McManus, Christopher J. Chuck

**Affiliations:** 1 Department of Mechanical Engineering, University of Bath, Bath, BA2 7AY UK; 2 Department of Chemical Engineering, University of Bath, Bath, BA2 7AY UK

**Keywords:** Techno-economic analysis, TEA, Single cell oil, Lipid

## Abstract

**Background:**

Heterotrophic single-cell oils (SCOs) are one potential replacement to lipid-derived biofuels sourced from first-generation crops such as palm oil. However, despite a large experimental research effort in this area, there are only a handful of techno-economic modelling publications. As such, there is little understanding of whether SCOs are, or could ever be, a potential competitive replacement. To help address this question, we designed a detailed model that coupled a hypothetical heterotroph (using the very best possible biological lipid production) with the largest and most efficient chemical plant design possible.

**Results:**

Our base case gave a lipid selling price of $1.81/kg for ~ 8,000 tonnes/year production, that could be reduced to $1.20/kg on increasing production to ~ 48,000 tonnes of lipid a year. A range of scenarios to further reduce this cost were then assessed, including using a thermotolerant strain (reducing the cost from $1.20 to $1.15/kg), zero-cost electricity ($ 1.12/kg), using non-sterile conditions ($1.19/kg), wet extraction of lipids ($1.16/kg), continuous production of extracellular lipid ($0.99/kg) and selling the whole yeast cell, including recovering value for the protein and carbohydrate ($0.81/kg). If co-products were produced alongside the lipid then the price could be effectively reduced to $0, depending on the amount of carbon funnelled away from lipid production, as long as the co-product could be sold in excess of $1/kg.

**Conclusions:**

The model presented here represents an ideal case that which while not achievable in reality, importantly would not be able to be improved on, irrespective of the scientific advances in this area. From the scenarios explored, it is possible to produce lower cost SCOs, but research must start to be applied in three key areas, firstly designing products where the whole cell is used. Secondly, further work on the product systems that produce lipids extracellularly in a continuous processing methodology or finally that create an effective biorefinery designed to produce a low molecular weight, bulk chemical, alongside the lipid. All other research areas will only ever give incremental gains rather than leading towards an economically competitive, sustainable, microbial oil.

## Background

Approximately 40 million tonnes of biodiesel is currently produced globally, with the majority still produced from first-generation feedstocks, such as rapeseed, soybean or palm oil [1]. Approximately 15% of the total amount of these oils produced is now used for biodiesel. However, these feedstocks compete with land for food production and in the case of soybean and palm with virgin rainforest. The climate and biodiversity impacts posed by the use of terrestrial crop oils like palm oil and soybean oil is being increasingly understood. One potential alternative is to harness oleaginous microorganisms as a direct oil substitute, reducing the amount of vegetable oil used for biodiesel production [2–4]. Single cell oils (SCOs), are accumulated intracellularly in oleaginous microorganisms, normally under nutrient limitation and carbon excess, in a process known as de novo accumulation. Microbial lipids are typically mainly triglycerides, with a fatty acid profile similar to terrestrial vegetable oils, which makes them an attractive option for a wide range of applications from food supplements to precursors in the chemical and biofuel sectors [5, 6]. Arguably, the most heavily researched SCOs are from microalgae grown phototrophically in large outdoor raceway ponds or in photobioreactors [7, 8], however, after numerous commercial failures and a large body of work modelling the cost and environmental impact [9], the majority of commercial and academic interest is now invested in heterotrophic processing [4, 10].

As such a large body of research has been invested in developing heterotrophic SCO, with over 100 publications in 2019 alone. Over 80 yeasts have been reported to produce lipid intracellularly (over 20% of the dry weight of the cell). The majority of the work published details the use of yeast lipids for fuel production, and as such there are numerous reviews detailing the suitability of SCO for biodiesel production [11–14], with the lipid profile of most oleaginous yeast being similar to rapeseed oil. This work has demonstrated that while heterotrophic processing is technically feasible, there are still important research gaps associated with both the economic and environmental viability. For the assessment of emerging technologies at the early stages of development, techno-economic analysis (TEA) is increasingly being used alongside life cycle assessment (LCA). This is something which is particularly common within US Department of Energy (DoE) research programmes. For example, a recent review of emerging technology assessment concluded that the use of TEA at an early technology stage  is very important [15]. The use of TEA in a prospective way at low technology readiness levels (TRLs) enables the early determination of product minimum selling price, as well as understanding the key variables which effect cost and ultimately commercial viability. However, there are a number of challenges associated with doing this which include: data availability, uncertainty associated with scale-up from the laboratory, and other general uncertainties typically handled through scenario and sensitivity analysis.

Laboratory research published over the last decade for heterotrophic processing has predominately focused on improving the lipid productivity. For instance, *Rhodosporidium toruloides* cultivated on glucose reached 127 g/L of cell density with 61.8% w/w lipid content and lipid productivity of 0.54 g/L/h under fed-batch mode, while it achieved similar oil content (60.4% w/w) and productivity 0.55 g/L/h under draw-fill cultivation [16]. The sugar conversion to lipids was 23 and 24% w/w, respectively. Higher cellular density of 185 g/L and 0.88 g/L/h lipid productivity were achieved by *Rhodotorula glutinis* on glucose, subjected to oxygen-enriched aeration [17]. A lipid productivity of 1.6 g/L/h was noted for the cultivation of *Lipomyces starkeyi* on glucose with a final 64.9% w/w oil content reported for this system [18]. Similar productivity, 1.2 g/L/h was achieved by the engineered yeast strain *Yarrowia lipolytica* on glucose [19]. Further work has sought to use inexpensive substrates with low or zero pre-treatment and optimisation of cultivation parameters, as well as lower cost downstream operations [20–22].

From a process engineering angle, most authors reason that since achieving high cell-density is a prerequisite for high intracellular product titres, advanced cultivation modes, such as fed-batch, draw-fill, continuous and two-stage fed-cultivations attain improved cell densities and consequently higher lipid titres [11]. Regulation of the feed rate and design of its composition delivers better carbon-to-nitrogen ratio, allowing the stoichiometric requirement of carbon flux for growth to generate lipid-free cell mass and the excess carbon to lipid synthesis [16, 23]. Multi-parameter optimisation [24, 25] and kinetic modelling approaches [5, 26] to identify the best operating conditions for high productivity are also under development. While there are limited reports of heterotrophic lipid production on the commercial scale [27], a number of papers have detailed pilot-scale cultivations of oleaginous yeasts at 50–300 L which provide some insight into the upscale performance [28, 29], including using semi-continuous processing [30].

Despite all these publications, reviews, perspectives and insights, there is no clear indication of whether a heterotrophic process is even economically feasible, especially in the replacement of lower value lipids necessary for biodiesel production. As opposed to algal modelling, only a handful of techno-economic studies have dealt with the design of a microbial lipid production plant, with predicted prices being between $1.72–$5.9/kg [27, 31, 32].

All of these studies demonstrated a far higher lipid selling price than that of conventional oils, discouraging progress on taking heterotrophic processes to scale, and from this it looks unlikely that SCO grown heterotrophically could compete with plant oils which are sold for between $ 0.5–1.9/kg [34, 35] (Table 1). All of these studies demonstrate that there are large cost impacts in the upstream (cost of raw materials), midstream (bioreactor-associated utilities) and as lipids are synthesised intracellularly, the need for cell disruption increases the complexity and costs of the downstream processing stages as well [36]. The sensitivity analysis demonstrates that rather than a single aspect, almost all parts of the process have a combined impact, demonstrating that there is no simple chemical engineering fix to reduce the price. However, in all of the above work, the processes are modelled on real microbes, with experimentally determined growth kinetics. Despite these studies being a suitable guide as to what could feasibly achieved with the current organisms and sensible plant design, they do not demonstrate which scientific advances in key areas would go the furthest in making SCO competitive.

**Table 1:** Prices for common vegetable oils in October 2019 ( source: Indexmundi [33])

Vegetable oil	Price ($/t)
Soybean	776
Rapeseed	909
Sunflower	771
Palm	591

Therefore, there is a key knowledge gap of what is absolutely the lowest price that lipid could be produced for. As there are biological limits to how much lipid can be produced from a unit of sugar and engineering limits to the scale that the lipids can be produced on, it is theoretically possible to determine this absolute minimum. This is vital in this area to determine firstly whether there is any point continuing research into SCO production for biofuels, and secondly what areas should be specifically targeted to reduce the price effectively. To this end, in this study, we use a novel approach to produce a prospective assessment, not to produce a techno-economic model that demonstrates what is plausible, or even what is feasible at this early stage, but one to investigate the *absolute lowest possible minimum lipid selling price* if a biological system was run to its theoretical maximum, in a chemical plant that was designed to be the most effective possible. This is the first time that this has been demonstrated for lipid production and the aim is therefore not to show at what lipids *could* be produced for, but what the price of lipid is where no possible scientific advance in heterotrophic cultivation could deliver a cheaper lipid, and by extension what areas in SCO production should be heavily researched as being the most effective way of producing a competitive SCO.

## Results and discussion

### Cultivation workflow description

In our hypothetical process, a single cultivation lasts for 28 days, with the batch growth phase lasting for 2 days and the rest dedicated to draw-fill operation with daily harvesting. In particular, at the end of the batch and every 24 h, 30% of the broth is removed and replaced with fresh medium. Addition of fresh medium creates gradients of pH, temperature and nutrient concentrations, which need to be absorbed as soon as possible to avoid stressing and lag phase of the microorganisms. At large scale restoration of the cultivation conditions cannot happen within minutes so the harvest/refill volume was limited to the 30% of the bioreactor working volume [30]. On the last day of the cultivation, the whole content of the reactor was emptied and processed. Cell concentration was assumed to be maintained at 185 g/L with an oil content of 60.4% w/w and the lipid profile was assumed to be similar to palm oil. The air flowrate was assumed to be 0.5 vvm. With an overall growth rate of 63.58 g/L/d, the 44.4 t/day of cells needed to level the concentration to 185 g/L are generated. The plant operates for 8400 h/years (350 days) in a 24-h basis and the production was designed based on the annual sugar supply (which we assumed to be stored after the growing season to allow all year-round operation of the plant).

The sugars obtained from the circular area in Sao Paulo are far in excess for use in one 1000-m^3^ reactor under these conditions and so do not limit the production for a single bioreactor system. Between each cultivation cycle, 3 h were allowed for cleaning-in-place and loading and uploading for 8 h each with a 100 m^3^/h rate. The time required to withdraw and refill the 30% of the broth was also taken into account. All unit operations downstream to the bioreactor were assumed to operate in continuous mode. From the lipid yield on sugars the required amount of sugar needed was calculated. The fermentation workflow and the cultivation details are depicted in Table 2.

**Table 2:** Operation workflow and microbe details

Yeast-related properties	
Mode of operation	Draw-fill
Maximum DCW (g/L)	185
Lipid content (% w/w)	60.4
Lipid productivity (g/L/h)	1.6
Temperature (°C)	30
Workflow details	
Working volume (m^3^)	800
Clean-in-place (h)	3
Uploading/unloading time (m^3^/h)	100
Fermentation (h)	672
Harvest volume (% v/v)	30%
Number of harvests	27
Plant operation details	
Plant operation (h/year)	8400
Number of fermentations/year	10
Run time per cycle (h/cycle)	820.6
Mass of microbial oil (t/year)	8052.47

### Detailed description of the plant equipment

**Fig. 1:**
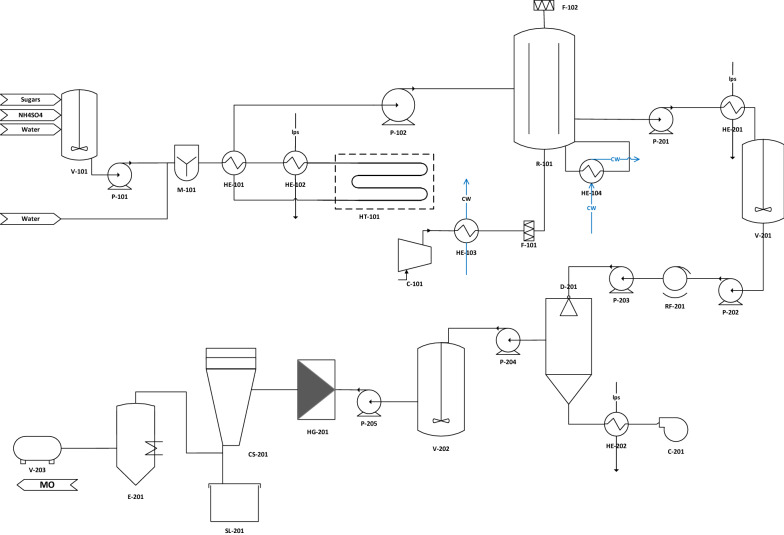
Process flow diagram (PFD) for the production of microbial lipids

The process flow diagram (PDF) for the process is depicted in Fig. 1. The process is structured in two areas, Area 100: upstream and cultivation (media preparation, sterilisation, bioreactor and associated utilities) and Area 200: downstream (cell harvesting and lipid recovery).

It was modelled so that the nutrients (sugars, ammonium sulphate and water) were mixed in a mixing tank V-101 to form a concentrated solution which was later diluted with the required amount of water to reach the final concentration of nutrients through an in-line mixer (M-101). It was calculated that the medium was then transferred to the continuous steriliser. The steriliser consisted of three parts [37]: HE-101 is the pre-heating section of the steriliser where the incoming media exchange heat with the sterile media going into the reactor which in turn are cooled to 30 °C, the heating section HE-102 where the media reach the sterilisation temperature 120 °C, which is maintained at the holding tube of the steriliser (HT-101), where the media remained for 2 min. The sterile media was then cooled down in HE-101 and are transferred with the aid of a pump P-102 to the bioreactor R-101.

23,000 m^3^/h of air was supplied through a centrifugal compressor C-101, necessary to supply the large amount of air required. The temperature of the broth was then maintained at 30 °C with external cooling via recirculation. The harvested broth passed through a pasteuriser HE-201, where the cells are deactivated for 60 min at 65 °C, this was put in to ensure stability through all downstream operations and storage. After pasteurisation the broth was kept in a set of holding tanks (in order to be processed at a suitable rate). The cells were then separated from the broth with a vacuum rotary filter, RF-201, which has a filtration rate of 100 m^3^/h. The yeast cake was then treated in a spray dryer (D-201), which dries the yeast paste from an assumed moisture content of 35% [38] to 5% [39]. After that the dried yeast was mixed with hexane in a mixing tank V-202 at a ratio of 25% yeast mass to hexane [27, 31] and then homogenised in a high-pressure homogeniser HG-201. The lysed cells were separated from the lipids and hexane in a centrifugal separator (CS-201), stored in a silo (SL-201) and then the hexane was recovered in a single-effect evaporation unit (E-201). The resulting lipids were then assumed to be kept for a short period of time in a holding tank (V-203), from where they are transported to the buyer.

### Single bioreactor microbial lipid production facility

From the circular area in Brazil 254,981.14 t/year sugars can be obtained. In the model for a single bioreactor 8052.49 t of microbial lipids are produced per year. The lowest cost of sucrose that was found in the literature was $ 0.14/kg and so this value was used throughout [28]. The equipment and utility costs associated to this capacity are presented in Tables 3 and 4 and the raw materials cost in Table 5. The FCI reached $16,085,855. The fermentation-related installed equipment cost was 36% of the total installed equipment cost in contrast to other works with more bioreactors. The airlift bioreactor itself constitutes 8% of the installed equipment cost. The bioreactor cooling requires a large amount of water as a cooling agent. To save water, the cooling water was modelled to be recycled after every cycle. The annual bioreactor cooling cost was calculated to be $76,382. If recycled, the cost of water is reduced to $6,365/year, saving from the cost of utilities $1,070,000, by using less water, than that without recycling.

**Table 3:** Equipment specifications, installed equipment costs and fixed capital investment

	Code	Type	Size		Units	Material	Number	f.o.b. ($, unit cost)	*F*_BM_	*C*_BM_ ($, unit cost)	*C*_BM_ ($)	Source the calculation was based on
Area 100 media preparation + fermentation	V-101	Mixing tank	200		m^3^	SS304	1	173,977	1.80	313,159	313,159	[40]
	A-101	Agitator	5.51		kW	SS	1	31,560	1.50	47,340	47,340	[41]
	M-101	In-line mixer	50		m^3^/h	SS316	1	41,271	1.70	70,161	70,161	[41]
	P-101	Centrifugal pump	29.41		kW	SS316	1	28,467.39	2.30	65,475	65,475	[32, 41]
	HE-101	Heat exchanger (shell and tube)	470.25		m^2^	SS314	1	295,594.85	3.29	972,507	972,507	[40]
	HE-102	Heat exchanger (shell and tube)	11.66		m^3^	SS314	1	23,326.98	3.29	76,746	76,746	[40]
	HT-101	Holding tube	L = 120 D = 0.4 Sch. 40 pipe		m m	SS314	1	308,766.0516	2	617,532	617,532	[42]
	P-102	Centrifugal pump	29.41		kW	SS316	1	47,137.35	2.30	108,416	108,416	[41]
	C-101	Centrifugal compressor	1468.46		kW	CS	1	1,240,647.12	1.60	1,985,035	1,985,035	[40]
	F-101	Filter	-		-	-	1	-	-	14,199	14,199	[32]
	F-102	Filter	-		-	-	1	-	-	93,561	93,561	[32]
	HE-103	Compressor cooler	28.90		m^2^	CS	1	21,291.23	3.29	70,048	70,048	[40]
	R-101	ALB (bioreactor)	1000		m^3^	SS316	1	-	-	991,615	991,615	[38]
	HE-104	Bioreactor Chiller	744.00		m^2^	SS316	1	458,438.06	3.29	1,508,261	1,508,261	[40]
Area 200 Downstream Processing	P-201	Pump (discharging)	185.19		kW	SS	1	29,585.37	2.30	68,046	68,046	[41]
	HE-201	Pasteuriser	16.39		m^2^	SS316	1	42,287.66	3.29	139,126	139,126	[31]
	V-201	Holding tank	400.00		m^3^	SS316	3	173,977.37	1.80	313,159	939,478	[40, 43]
	A-201	Agitator	5.51		kW	SS	3	35,816.69	1.50	53,725	161,175	[41]
	P-202	Pump	80.00		m^3^/h	SS316	3	45,432.38	2.30	104,494	313,483	[41]
	RF-201	Rotary vacuum filter	200.00		m^2^		1	414,134.36	1.4	617,428	617,428	[31, 44]
	P-203	Pump	148.15		kW	SS316	1	28,467.39	2.30	65,475	65,475	[40]
	D-201	Spray dryer				SS316	1	-	-	1,110,098	1,110,098	[39]
	P-204	Pump	29.41		kW	SS316	1	28,467.39	2.30	65,475	65,475	[41]
	C-201	Fan	17.54		kW	CS	1	16,156.00	1.60	40,088	40,087.79	[42, 45]
	HE-202	Air heater	105.39		m^2^	SS316	1	44,020.54	3.29	144,828	144,827.56	[40]
	V-202	Mixing tank	100		m^3^	SS316	1	166,792.77	1.80	300,227	300,227	[40]
	A-202	Agitator	5.51		kW	SS	1	31,560.16	1.50	47,340	47,340	[41]
	P-205	Pump	29.41		kW	SS316	1	28,467.39	2.30	65,475	65,475	[41]
	HG-201	Homogeniser	45		m^3^/h	SS316	1	184,065.28	2.06	429,432	429,432	[46]
	P-206	Pump	29.41		kW	SS316	1	28,467.39	2.30	65,475	65,475	[41]
	CS-201	Centrifugal separator	0.686		m^3^/h	Ss316	1	442,391.50	1.3	575,109	575,109	[39, 46]
	E-201	Evaporator (single effect/agitated film, scrapped wall)	20.66		m2	SS	1	293,102.88	2.50	732,757	732,757	[40]
	SL-201	Concrete silo	1500		m^3^	Concrete	1	88,294.62	1.70	150,101	150,101	[41, 46]
	V-203	Holding tank	800		m^3^	SS304	1	244,559.38	1.80	440,207	440,207	[40]
FCI (1.2xC_BM_)											16,085,855

**Table 4:** Overview of utilities and labour costs

Equipment	Number of equipment	Workers/shift^a^	Electricity ($/y)^a^	Low-pressure steam ($/year)^a^	Cooling water ($/year)^a^
V-101	1	0	-	-	-
A-101	1	0.5	1028.53	-	-
M-101	1	0.3	3360	-	-
P-101	1	0	5489.87	-	-
HE-101	1	0.1	-	-	-
HE-102	1	0.1	-	72,616.55	-
HT-101	1	0.1	-	-	-
P-102	1	0	34,567.90	-	-
C-101	1	0.1	274,113.24	-	-
F-101	1	0	-	-	-
F-102	1	0	-	-	-
HE-103	1	0.1	-	-	1105.07
R-101	1	0.5	-	-	-
HE-104	1	0.1	-	-	6365.18
P-201	1	0	34,567.90	-	-
HE-201	1	0.5	-	748,448.88	-
V-201	3	0	-	-	-
A-201	3	0	3085.6	-	-
P-202	3	0.2	82,962.96	-	-
F-201	1	0	-	-	-
P-203	1	0.2	27,654.32	-	-
D-201	1	0	4704	-	-
P-204	1	0.2	34,567.90	-	-
C-201	1	0.1	689,747.40	-	-
HE-202	1	0	-	698,730.10	-
T-202	1	0.5	1028.53	-	-
A-202	1	0	-	-	-
P-205	1	0	1028.53	-	-
HG-201	1	0.3	5489.87	-	-
P-206	1	0	5644.8	-	-
CS-201	1	0.25	5489.87	-	-
E-201	1	0.3	-	-	-
SL-201	1	0	-	1,168,927.40	-
T-203	1	0	-	-	-
Total ($/y)		450,000	1,184,425	2,688,723	7470

^a^The costs are related to the total number of equipment, not the unit cost

**Table 5:** Summary of the raw materials amounts and costs

Raw material	Amount (t/year)	Unit cost ($/kg)	Total cost ($/year)	Ref
Sugars	32,208.00	0.14	4,509,120	[28]
Ammonium sulphate	646.20	0.16	103,392	[32]
Hexane	3907.2	0.41	1,602	[31]
Total			4,614,114	

Similarly, the hexane required for the lipid extraction was assumed to be recycled after each cycle. To design the bioreactor size, the aspect ratio was considered in respect to the diameter and height impact on the aeration rate. A larger diameter would require larger aeration rate, while larger height creates higher hydrostatic pressure. In its turn the hydrostatic pressure determines the size of the compressor. By consulting sources regarding to the scale of the airlift bioreactor used in ICI’s Pruteen process, the bioreactor parameters were set as such (*h*: 55.5 m, *r*: 2.4 m) to allow a hydrostatic pressure of 4.25 atm, for which a compression ratio of 4 means only one compressor is needed. The compressor was sized accordingly to overcome the hydrostatic pressure and was calculated to deliver 23,952.10 m^3^/h of air at a discharge pressure of 4.25 atm.

Initially a continuous system was also examined, however we considered it is highly unlikely that the maximum cell concentration, used in the draw-fill case, could be maintained with this system, therefore with a lower cell concentration, the lipid production would be similar or worse than the draw-fill case [30]. Continuous processing is normally applied to processes producing extracellular molecules that can then be stripped from the broth, in the case of lipid production this is technically feasible with the latest advances in metabolic engineering, and therefore was addressed in the latter scenario.

### Economy of scale

For a plant containing a single bioreactor only, the lipid selling price calculated was $1.81/kg. This is comfortably lower than the estimated prices for the more realistic models presented in the literature [27, 31]. However, the total amount of sugar used is still 7 × less than can be feasibly collected in an area around the plant. As such the effect of economy of scale was assessed for up to 7 airlift bioreactors. For simplicity of calculations the equipment and materials associated with the bioreactor were modified. In particular, the bioreactor number was increased from 1 to 7 along with the air filters, the compressor and its respective cooler, the bioreactor chiller, the pasteuriser and the holding tanks to regulate the downstream processing of deactivated cells were modified accordingly.

**Fig. 2:**
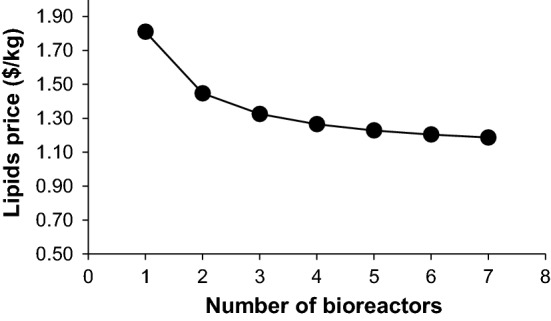
The impact of economy of scale on the price of lipid ($/kg)

Unsurprisingly, the lipid production price changes dramatically with an increased economy of scale (Fig. 2). While the equipment cost increases, the multipliers for FCI and COM absorb the increase in installed equipment cost and even though the utilities are greater, in conjunction with the larger oil production, the price decreases. There is little benefit to increasing beyond 6 bioreactors, and so this was selected as the appropriate largest feasible size of plant. This is a reasonable assumption since previous techno-economic works modelled 10 to 12 stirred tank bioreactors, ranging from 250 to 750 m^3^ to achieve the targeted annual production.

The share of these bioreactors together on the total equipment cost ranged from 68 to 90% [31, 32, 46]. What is more, a breakdown of the electricity used for the bioreactor showed that it was 53% of the total electricity cost [31]. In this study, the cost of one airlift bioreactor was set as $991,615, lacking of agitator and electricity costs, therefore not responsible for the biggest impact on the installed equipment cost. The volume modelled is larger than those usually studied and the strain is highly productive. Table 6 shows how the cost of manufacture, FCI, raw materials and utilities are affected by the increased bioreactor number and lipid amount.

**Table 6:** Summary of lipids price, equipment, utilities cost and raw materials for different bioreactor numbers

Reactor number	Production (t/year)	COM ($)	Oil price ($/kg)	FCI ($)	COL ($)	CRM ($)	CUT ($)	Sugars needed (t/year)
1	8053	14,572,474	1.81	16,085,855	450,000	4,614,114	3,880,618	32,208
2	16,105	23,303,062	1.45	23,626,689	556,875	9,228,227.11	5,023,796	64,416
3	24,157	32,018,294	1.33	31,167,523	658,125	13,842,340.67	6,166,975	96,624
4	32,210	40,733,526	1.26	38,708,357	759,375	18,456,454.22	7,310,154	128,832
5	40,262	49,448,758	1.23	46,249,191	860,625	23,070,567.78	8,453,332	161,040
6	48,315	58,163,990	1.20	53,790,025	961,875	27,684,681.34	9,596,511	193,248
7	56,367	66,879,221	1.19	61,330,859	1,063,125	32,298,794.89	10,739,689	225,456

### Comparison to alternative techno-economic studies

In one of the original, most detailed studies Koutinas et al*.* reported prices of $5.5/kg oil and $5.9/kg for biodiesel for a process that used $400/t of glucose and produced 10,000 t/year of oil [31]. They modelled this using 12 × 250 m^3^ stirred tank reactors and found that indirect transesterification of lipids to biodiesel was more economical than direct transesterification. Similarly, Braunwald et al. [32], compared 750-m^3^ stirred tank bioreactors to 1260-m^3^ open ponds for an oleaginous yeast cultivation and estimated that in the first case the price was $2.35/kg with the fermentation, harvesting and drying costs contributing to the 87% of the total cost, while the open ponds were cheaper at $ 1.72/kg with 43% contribution to the cost. Despite the higher experimental cell and lipid yields used in the study of Koutinas et al. [31], the cost estimation of the latter study was lower, probably due to the larger bioreactors used. More recently, techno-economic assessment of microbial lipids at different scales (100 t/year and 10,000 t/year), using lignocellulosic feedstocks was assessed. This was modelled for 12 stirred bioreactors (250 m^3^) and compared to open ponds [27] to assess the variability of capital expenditure and minimum selling price according to scale and various scenarios. For the larger production facility, lower lipid selling price was noted for sucrose ($4.64–5.41/kg) and wheat straw ($5.15–5.41/kg) while the pre-treatment required to increase the carbon content of rich feedstocks such as distillers dried grains contributed to the upstream cost.

The lowest estimated price presented to date has been for an integrated refinery concept which assumed a selling price of $1.3/kg for lipid and $0.5/ kg for the defatted biomass, calculated for a single-cell oil produced from molasses at $99/tonne, as part of an integrated refinery with sugar production. This was calculated by modelling exponential fed-batch fermentations with 11 × 500 m^3^ stirred tank bioreactors [46]. The latter lower price was achieved through replacing the stainless steel bioreactor with the cheaper alternative of carbon steel vessel with epoxy lining to reduce the capital cost and by combining in a sugar mill, reducing the cost of the molasses substantially [46]. Though this study lacked the full detail of the previous studies, it is a useful indicator that valorising the defatted biomass can aid in the reduction of the overall lipid price.

In a more recent publication by Koutinas et al., the group produced a slightly lower oil price of $4.613/kg for 10,000 t/year production of lipids and estimated the price would be between $5.8 for 2000 t/year and $4.1/kg for 40,000 t/year production capacity [47]. Their minimum lipid selling price of $2.5/kg was estimated for the case of $0.10/kg glucose, which is similar to the price of sugars used in this work ($0.14/kg) and close to the lipid price of the single bioreactor facility, $ 1.81/kg for ~ 8000 t annual production.

In contrast to all previous studies, this work based the cost estimations on one very large bioreactor, assuming ideal yields and an optimal conversion process. As a cost-saving approach, the bioreactor was airlift, while other works used more than ten conventional stirred tanks. Subsequently, the estimated lipid price was lower than that of other techno-economic analyses. The lipid price of $1.82/kg was around 3 times lower than that of Bonatsos et al*.* [47] and Parsons et al*.* [27] but was closer to the open ponds model ($1.72/kg) [32]. The similarity of the prices indicates that vessels with low running costs have a big impact on reducing the production cost.

### Alternative processing scenarios

The lowest cost of lipid is reduced to $1.20/kg using 6 bioreactors, a 33.7% reduction in the price. However, a number of other scenarios have been presented in the literature, which have claimed to reduce the price of lipids substantially. To investigate these claims, a range of scenarios were assessed for the effect on the lipid selling price, these included having access to inexpensive electricity, using a non-sterile process, using a thermotolerant species, using a species that could produce the lipid extracellularly, using wet cell extraction and removing the extraction stage altogether and selling the lipid and cell as one product.

As seen previously, due to the lipids being an intracellular product, there is a range of recovery steps, from which some are costly in terms of equipment and energy consumption. In order to achieve above 95% lipids recovery and above all steps from cells filtering, drying and disruption [48] should be efficient. The proposed process was reviewed and edited by removing specific downstream steps and consider an alternative end-use for the lipids and/or biomass together.

### Effect of electricity price on the lipids cost

**Fig. 3:**
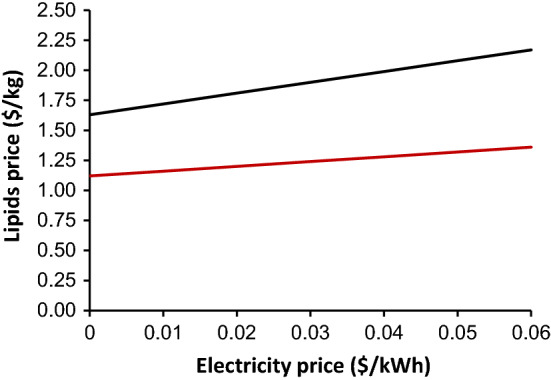
The impact of electricity prices ($/kWh) on the selling price of lipids ($/kg) for the one bioreactor scenario (black line) and six bioreactor scenario (red line)

The production of single-cell oil is a high energy process, and as such the cost of electricity has been cited as a major cost contributor in microbial oils production [31]. In this work, the lowest possible price of electricity for industrial use was used, $0.02/kWh [49], without necessarily being the cost of electricity in Brazil. To investigate how different prices of electricity affect the selling price, the latter was modelled for prices ranging from $0.00–0.06/kWh. In this model the lipid price was found to increase by approximately $0.1/kg for a $0.01/kWh rise in electricity price. Sugarcane bagasse is burned to satisfy the energy requirements of sugar mills and 36.7 kWh of electricity can be generated from a tonne of crushed sugarcane [50]. Design works on bioproduction plants, similarly adjacent to sugarcane mills, consider burning bagasse for electricity generation for increasing revenue or for use in the mill and investigate combined heat and power (CHP) to increase efficiency [51]. Therefore, the surplus electricity from the mill can be directed to cover part or the whole of the electricity demand of the microbial lipids plant, reducing an important cost contributor. It was envisaged that the electricity can be obtained for free if it is subsidised or produced internally. In our presented scenarios, the minimum price reduces to $1.63 /kg for the single bioreactor base case process with zero cost of electricity, however, for the six bioreactor scenario the price is not reduced substantially and the lipid still costs $1.12/kg with no electricity cost (Fig. 3).

### Non-sterile conditions

A few experimental works have explored the potential of non-sterile cultivation of oleaginous yeasts to reduce the cost by sterilisation at large scale [52, 53]. Maintenance of monoculture can be facilitated by adoption of harsh culture conditions, such as low pH or addition of toxic compounds, selective to the target microorganism. To adapt the model to this hypothetical scenario, the continuous steriliser was removed with its associated steam requirements and it was assumed that the organism was able to secrete antimicrobial compounds and survive in low pH, as previously reported by Santamauro et al*.* [54].

**Fig. 4:**
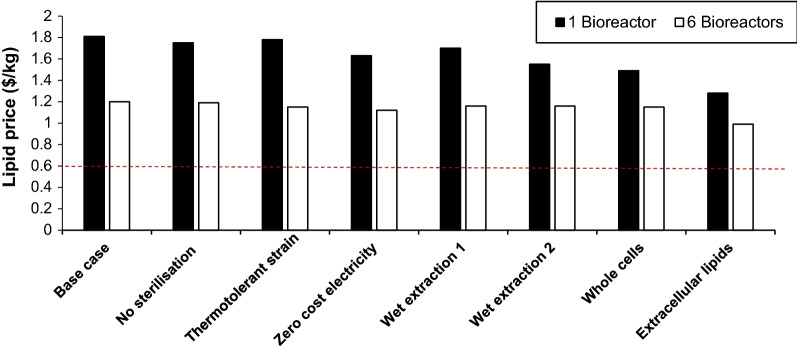
The impact of alternative processing scenarios on the lipid price ($/kg) for the one bioreactor scenario (black) and six bioreactor scenario (white), the red line shows the palm oil price (Nov. 2019). Wet extraction 1 is the case where extraction and stripping columns were used, while wet extraction 2 is the case where the spray dryer was removed

Removing sterilisation affects 12% of the installed equipment cost, 11% of the operating labour and only 2% of the total low-pressure steam cost. Its removal drops the production cost of lipids from $1.81 to $1.75/kg, for the one-reactor scenario and from $1.20 to $1.19/kg for the six-reactor scenario (Fig. 4). This is a modest saving, however in addition, it should be noted that removing sterilisation altogether is a rather controversial modification, as possible hardy contaminating species entering with the media will be difficult to get rid of, especially at such large scale. The main microorganism needs to be really robust to remain the dominant population and if the lipids are used in the food industry, relevant regulations would be difficult to meet. It is unlikely that this is a plausible scenario at all, but rather these ultra-robust organisms act as another buffer against contamination alongside conventional strategies.

### Thermotolerant strain

In a similar vein to the non-sterile scenario, thermotolerant microorganisms are an attractive option for bioconversions taking place in environments with higher ambient temperature or for withstanding a rise in the broth temperature due to exothermic metabolic reactions and agitation [55]. By using a microbe able to tolerate higher operating temperatures, the need for cooling is reduced, followed by energy savings and reduction of cooling water requirements for the bioreactor. To apply this idea in this process, the chiller and its water requirements were removed. However, elevated temperatures reduce the dissolved oxygen concentration which would also, in reality, reduce the productivity of the yeast. The increase in the evaporation effect, phenomenon preferable in ethanol production as it can be stripped out of the broth more easily, would only increase the amount of water needed for the bioreactor in this case. However, not taking these factors into account, by omitting the chiller, there is a 11% saving in installed equipment cost but 85% on the cooling water requirements, as in order to cool a broth of 800 m^3^, large amounts of water were needed. Nevertheless, the impact on the price of lipids is actually quite low, with the price of the SCO from one reactor dropping to $1.78/kg and for 6 bioreactors only being reduced to $1.15/kg for the six bioreator scenario (Fig. 4).

### Wet cell lipid extraction

Drying is a costly process as there is a need for an air fan and an air heater to provide the air and heat it to temperatures as high as 150 °C to dry the cellular paste. Wet lipid extraction has been considered in algal cells [56], where drying and homogenisation are omitted and the lipid extraction and separation take place in an extraction column followed by a stripping column.

Implementation of these stripping columns in this work raised the FCI to $29,818,363 and the working capital at 72% of the one bioreactor scenario for dry cells extraction. However, due to the way the COM is calculated (Eq. 4), the increase in FCI does not greatly affect the price of lipids, which is comparable to the dried extraction process (1.70/kg). The cost of raw materials altogether remains stable at around $4,615,000 as more hexane is now needed (1.7 times more than that used for extraction from dried cells as suggested by the method) according to the NREL process (Table 7) [56]. If the columns are not implemented but only the drier is removed from the base scenario and the rest of the process remains the same downstream of the dryer, the cost drops to $1.55/kg. That indicates that the drying step and disruption has a greater impact on the lipids price. When using 6 bioreactors, the price of lipids is $1.16/kg if extraction and stripping column are implemented, this is the same as when only the dryer step is removed.

**Table 7:** The impact of alternative downstream operation sequences on the process cost and lipids price

Scenario	FCI ($)	CUT ($)	CRM ($)	COL ($)	COM ($)
Base scenario	16,085,855	3,880,618	4,614,114	450,000	14,572,474
No sterilisation	14,085,713	3,808,001	4,614,114	425,000	14,054,880
Thermotolerant strain	14,275,942	3,946,869	4,614,114	450,000	14.328,178
Zero-cost electricity	16,085,855	2,696,193	4,614,114	450,000	13,115,631
Wet extraction 1	29,818,363	1,398,219	4,615,182	325,000	13,651,038
Wet extraction 2	14,453,269	2,532,399	4,614,114	400,000	12,483,798
Use of whole cells	12,718,507	2,694,037	4,612,512	275,000	12,027,137
Continuous extracellular lipids	19,145,521	1,295,366	104,669	300,000	10,318,648

### Use of the whole microbial mass as a lipid, protein and nutrient source

Apart from the lipid droplets, the cell mass contains nutritious molecules, such as carbohydrates and proteins. Oleaginous yeasts were originally grown for their protein content [6] and use of oleaginous biomass produced for aquafeed has also been reported [57]. There has been a growing interest in producing microbial feed ingredients as animal feed additives, using bacteria and yeasts [58]. Using intact cells as a feed ingredient, takes away a large part of the recovery process and most importantly the need to use solvent to extract the lipids. The mixing tank with the hexane, the homogeniser and its electricity, the evaporator and the decanter centrifuge, the low-pressure steam and the labour cost were therefore removed from this scenario. The pasteurising and drying steps are maintained as the first will ensure the cells are not active while the latter will allow for increased shelf life. The cost savings from this process are 21% in installed equipment cost, 34% in labour cost, 66% in utilities and $1,601 from omitting hexane from the cost of raw materials.

The overall reduction in the price of lipids drops to $1.49/kg, 17.6% cheaper than the base process for the one-reactor scenario. This case has value in terms of reducing downstream processing and steps that can compromise the extraction efficiency or affect lipids quality while it removes the need for further treatment and disposal of the defatted cells upon extraction as previously. When using 6 bioreactors the price of lipids drops to $1.15/kg (Fig. 4). This is calculated as if the protein and carbohydrate have no value attached to them, and would only really be suitable in the food and surfactant sectors, rather than for fuels.

### Development of a continuous process of extracellular lipid production

If lipids could be produced extracellularly, drying and cell disruption would be unnecessary and the efficiency recovery could be extremely high. Extracellular release of lipids has been reported for yeasts cultivated in acetic acid-media [59, 60]. *Cryptococcus curvatus* released lipids to the broth when cultivated in media containing more than 20 g/L acetic acid. Work in the same research group investigated further this phenomenon, which is a result of compromised integrity of the cellular membrane when subjected to elevated concentrations of the acid [59] and is now the subject of experimental optimisation as an attractive option for lipids recovery [61]. Further to this work, interesting steps have been taken with a genetically transformed *Y. lipolytica*, that was able to produce lipids extracellularly [62] and the bacterium *Escherichia coli* which has been engineered to release fatty acids [63].

To determine this effect, extracellular lipid production was investigated here by assuming that the yeast culture could be held at maximum biomass (185 g/L) for 28 days at a time, and thereafter converting the sugars solely to triglyceride with a weight conversion of 32% (the molar theoretical maximum). For the recovery of extracellular lipids, a major part of the conventional downstream operation of the proposed process was not required. The cells were assumed to be separated from the broth with a rotary vacuum filter and the supernatant further processed through sedimentation in a mixer/settler, where lipids are separated from the rest of the broth due to density differences. Sedimentation of lipids has been recently reported for recovering sophorolipids at high efficiency [64]. This method reduced the cost of 1 reactor to $1.28/kg and for the 6 reactor scenario to $0.99/kg (Fig. 4).

### Reducing the cost of single-cell oils through a biorefinery concept

Further product valorisation is possible under a biorefinery concept, where all by-products are considered valuable and commoditised. In the first instance, if the lipid extraction process is followed, the defatted cell mass is also a side stream that has value. The lipid-free mass contains proteins and carbohydrates and can be recycled to the fermentation as a yeast extract alternative in the same process [65, 66], converted to methane in an anaerobic digester [56] or used as additive to animal feed [31]. For a set revenue of $14,572,474 if spent cells were valued at $0.6/kg (the same value given by Parsons et al*.* [27]) the lipid price could further drop to $1.42/kg for 1 bioreactor from $1.81/kg, while for 6 bioreactors the price drops to $0.81 from $1.20/kg.

**Fig. 5:**
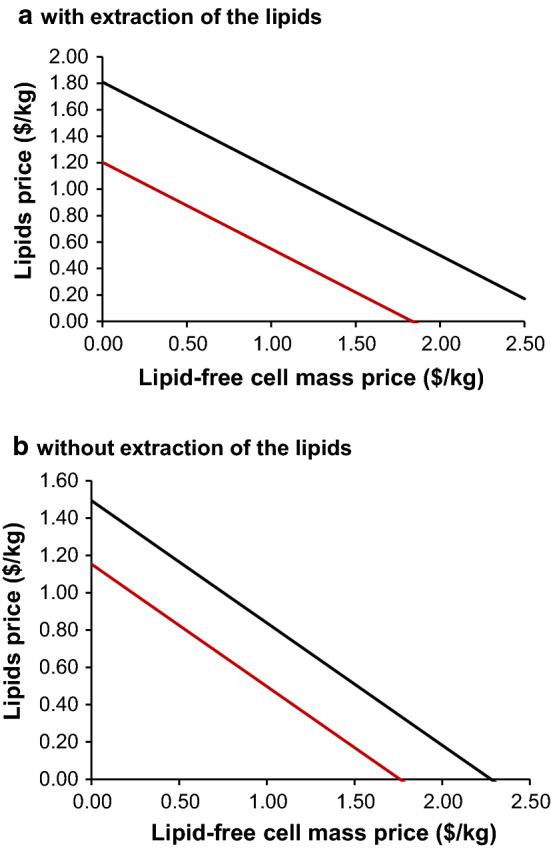
Impact of the price of lipid-free cells mass ($/kg) on the lipids price **a** with lipid extraction for the one bioreactor (black line) and six bioreactor scenario (red line), **b** without lipid extraction when lipid-free cells and lipids are priced separately but sold together as the whole cell, for the one bioreactor (black line) and six bioreactor scenario (red line)

In other reports, spent cells have been valued at anywhere between $0.5–2.5/kg and have been demonstrated to increase the revenue from microbial oil production [27, 31, 46]. Higher revenue is achieved when spent cells are used as animal feed compared to energy generation and can counterbalance other process expenses, such as the cost of raw materials [31]. To investigate this effect, two scenarios were used, where the lipid is extracted from the spent cells and the lipid and spent cells are sold separately versus where the whole cell is sold, without extraction of the lipid, but the non-lipid cell biomass also commands value (Fig. 5). The revenue was held constant, to assess the effect of the increased price of the biomass on the lipid price. Interestingly, the price of the lipid can be reduced substantially, even to $0, if a high enough value for the defatted biomass can be obtained. This demonstrates that a plant producing high-value cell biomass, with lipid as a co-product, could well produce a lipid that competes with palm oil.

A large proportion of oleaginous yeasts are able to produce other small molecules commonly secreted extracellularly. For example, some oleaginous yeasts have been reported to secrete citric acid, concurrently with lipid accumulation and even at larger titres than oil [67–69]. Similarly, other acids from the TCA cycle can be released to the broth [70, 71], pigments from red yeasts [72], 2-phenylethanol [73] and succinic acid [74] have all been reported in literature. If the diversion of carbon from the original sugar source is understood and developed appropriately for scale-up, a valuable product could be obtained adding an additional revenue stream to lipid production.

In this scenario, a generic co-product is assumed with a variable price ($0–3/kg), 40% carbon by weight (as common acids, such as succinic acid, citric acid and lactic acid contain ~ 40% carbon) and the total amount of sugar used in the system was held constant. The total carbon flux was therefore used to calculate the change in the system, with the carbon directed to co-product reducing the amount of yeast biomass and lipid produced from the system (and reducing the CO_2_ produced as a consequence). For example, in the base case with no co-product, 25% of the carbon goes to lipids, 41.4% total biomass (with 16.4% to lipid-free cells), 8.6% of the carbon remains unused and 50% is converted to CO_2_. In the co-product case, 0–50% of the original carbon was considered to go to the co-product, taking equal amounts of conversion from the maximum total biomass conversion and from the CO_2_ for each yield.

**Fig. 6:**
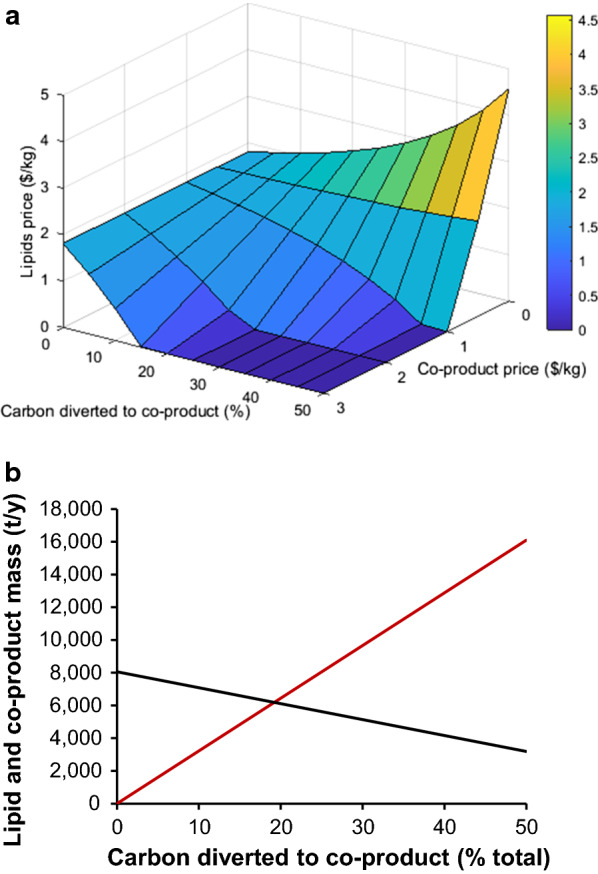
**a** Variation of the lipid price ($/kg) with concurrent co-product generation as a function of the amount of carbon diverted from cell production to co-product. **b** the impact of co-product generation on total lipid (black) and co-product (red) mass, where the co-product is assumed to contain 40 wt% carbon

To calculate the change in the lipid price, the annual revenue of $14,572,474 (where no co-product is produced) was held as constant. The price of lipids was calculated again for different co-product prices ranging from 0.5–3 $/kg, each for the different yields from 0 to 50% (Fig. 6).

If the co-product is valued at 0.5 $/kg, the increased co-product production, and subsequent reduction in lipid production increases the lipid price slightly, this is because that even though there is less carbon in the co-product than lipid, $0.5/kg is just not enough revenue to compensate for the loss in the higher value lipid. On the contrary, for co-product prices from 1 to 3 $/kg, the lipids price reduced with decreasing volume, in the most extreme cases the lipid price reaches negative values. This increases the process profitability and it means that lipids are produced for free, along with the co-product, which is now the main product while lipids would be considered as a co-product.

While the extraction of the co-product was not taken into account, as this would be highly dependent on the specific properties, this scenario demonstrates that it would be possible for SCO to compete with terrestrial lipids, if a smaller molecule, with less carbon in was produced alongside the lipid. There is an interesting question here, about whether a full commercial process would want carbon diverted to lipid production, if it could be used in a higher value product, but this could be a viable method of economic lipid production if mandated through policy or if the co-product can simply not be produced in large enough quantities to dominate the production process. However, the practicality of matching the scale of both products needed would be extremely challenging.

## Conclusions

Single-cell oils have the potential to replace terrestrial crops as a feedstock for next-generation biofuels, however, there is a huge uncertainty over whether SCO could ever compete economically with the current state of the art. In this study, the theoretical lowest possible lipid price was determined, by assessing a system to produce oil that is limited only by the amount of feedstock possible to be grown, the biological limitation of a heterotroph and the most efficient chemical plant possible. This still gave a lipid price approximately 2–3 times higher than palm oil. While it is not feasible to achieve these costs in reality, it would be an engineering impossibility to achieve a cheaper lipid product directly. Further reductions in the price are still possible however, but only through the co-production of a low molecular weight side-product and either the extracellular production of the lipid or the use of the whole cell in a product, though this has limited application for the fuel market.

The point of this paper is therefore to manage expectations in this area, the clear implication for scientists, engineers, policy makers and political decision-makers is that while reported systems could not actually economically compete with terrestrial oils, here we demonstrate that SCO could start being more competitive as long as more research effort was invested in a targeted approach directed towards these three areas as a matter of priority.

## Materials and methods

### Selection of carbon source and plant location

Sugars are a common carbon and energy source for microbial cultivations and oleaginous heterotrophs can consume them simultaneously or sequentially [75]. Using sugar crops for microbial oil production is simpler than starchy or cellulosic biomass, as pre-treatment and saccharification are not required [76], as such the cheapest source of sugars are still first-generation sugarcane [27]. As such our prospective microbial lipid production plant was assumed to be adjacent to a sugarcane mill from which sugars from sugarcane juice are provided. Hence, lower logistic costs and easy feedstock accessibility facilitate operations. The facility was hypothetically located in the state of Sao Paulo in Brazil, the largest sugar supplier [77]. Sao Paulo hosts the 60% of Brazilian sugarcane cultivation and accommodates a high density of sugarcane mills [78]. Sugarcane juice contains around 15% sugars [79] and the juice resulting from the milling process is usually concentrated in order to increase its shelf life prior to fermentation. Data on sugarcane yield and sugar content for the year 2018 were obtained from the relevant USDA report [79] and Raizen’s annual report [80]. We imagined that the facility lies in the middle of a circular area of 314 km^2^ (314,000 ha), of which 10% was allocated to industrial facilities in the centre, including roads and storage facilities, while the remaining 90% (282.6 km^2^) was allocated to sugarcane cultivation (Table 8). This is similar to the system described by Santos et al*.* for bioethanol production [81]. For this current work processing of sugarcane, extraction and storage of sugars were not considered, and rather were assumed to be in the original price of the sugar. This figure gives a maximum amount of sugar that can be consumed and therefore acts as the limitation on the scale of operation.

**Table 8:** Hypothetical annual sugar production from the implicit cultivation area (based on USDA yield values [79])

Parameter	Value
Area with sugarcane plantations (km^2^)	282.6
Yield of sugarcane (t/ha)	65.61
TRS/sugarcane (kg/t)	137.52
TRS extracted (t/year)	254,981

Assuming 100% harvesting efficiency in all steps

### Microbial species

The literature was reviewed to assign properties to a hypothetical, efficient oleaginous strain. The resulting strain was a blend of properties from several yeasts and was set as an optimistic example to define the best microbial factory. The overall achievable dry cell weight (DCW) was set as 185 g/L [17] with a 60.4% w/w oil content [16] and 1.6 g/L/h lipid productivity [18]. For the sugars-to-lipids conversion the practical maximum yield 25% w/w was adopted [82], while lower than the theoretical maximum yield of 32%, the best literature values tend to cluster in the range of 22–25% as higher yields are biochemically unfeasible due to carbon diversion to cellular growth and other metabolic routes. To satisfy a carbon balance of 100%, a 50% w/w of carbon was assumed to become CO_2_ based on calculations on the produced CO_2_ moles from glucose reported by Davies [38], while 16.39% of carbon is assigned to lipid-free cell mass. In the base case, no other product was produced from the cultivation with 8% of the carbon from the original sugars being unconsumed (in line with the majority of studies in this area). Lipids were accumulated according to the following equation:

$$0.232C_{6} H_{12} O_{6} + 0.27O_{2} + NH_{4} SO_{4} \to 0.78C_{4} H_{6.5} O_{1.9} N_{0.7} + 0.012C_{57} H_{104} O_{6} + CO_{2} + H_{2} O + 0.27SO_{4}^{ - }$$

### Bioreactor design

The operation of bioreactors is complex since sterility is required and, to achieve high oxygen transfer rates, high agitation and aeration rates are needed. High power demand is needed for the function of the agitator and the air compressor [83]. For larger reactors, larger agitators and moving parts are required and that is translated to higher power per unit volume required to achieve the desired oxygenation levels. As such, anaerobic fermentation vessels are significantly larger than those for aerobic processes, with anaerobic bioprocesses reaching volumes of 3,785 m^3^ and production of 2.5 billion litres of ethanol have been reported [80]. Typically, the number of small and medium size fermenters is larger for an aerobic plant than that of an anaerobic ethanol plant. Likewise, drying and centrifuging the cells are linked to significant electricity consumption during the separation processes [32].

The aeration and agitation needs for aerobic cultivations limit the operational maximum size of stirred tank bioreactors. These large stirred tank reactors are accompanied by higher energy requirements due to the need for aeration, agitation and function of large moving parts [84]. Besides that, bubble column bioreactors (BCR) are less expensive to operate and have 10–20% lower aeration costs than equal size stirred tank bioreactors (STR) [83]. Moreover, according to Humbird et al*.*, there is little benefit in scaling-up between 500-m^3^ and 1000-m^3^ BCR bioreactors, there is less manpower needed for larger vessels while the range of 500–1000 m^3^ is suggested for BCR reactors and a maximum of 500 m^3^ for STR [83]. In a similar screening exercise, 750-m^3^ and 1000-m^3^ bubble column bioreactors have been previously reported by NREL as being the most efficient with no advantage in increasing the size due to the issues of stability for taller reactors and the lower aeration afforded [85].

In this study therefore, to exclude agitator parts from the energy and cost calculations, a 1000-m^3^ airlift bioreactor (ALB) was employed, selected as the hypothetical largest column possible, while still retaining the benefits of the airlift system. The airlift has advantages over the bubble column, as a result of the presence of the riser and downcomer, such as prevention of bubble coalescence, more uniform flow pattern over the focal distribution of energy in the bubble columns and better heat and mass transfer efficiency [86, 87]. This system was also selected to reduce capital costs substantially over the suggested 12 stirred tank vessels reported previously.

### Mode of operation

Draw-fill cultivation has been applied to oleaginous yeast cultivations, and has achieved the highest cell culture concentrations [16, 30]. Humbird et al. assumed that the oxygen transfer rate should be equal to the oxygen uptake rate and suggested that for optimal aeration cost efficiencies when using bubble column bioreactors the range 50–150 mmol/L of OUR should be targeted and found that there are larger savings at lower OUR values [83]. Therefore, the broth was assumed to be non-viscous and for efficient operation of the airlift an oxygen uptake rate (OUR) of 50 mmol/L was chosen as this is at the lower end of the above range, but it is at the upper end for reported experimental studies. For all calculations, physicochemical properties of water were adopted for the fermentation media and broth, as they are aqueous solutions of the nutrients.

### Estimation of cost of manufacture

A ‘study estimate’ methodology (accuracy ± 30%) was used for the estimation of the capital expenditure and operating cost [88]. First, the properties of each process stream were defined and the required equipment, such as a heat exchanger or a pump, was designed as such to operate according to these properties. In particular, each equipment purchase cost (‘free on-board cost’ or f.o.b.) was calculated from equations of the form of Eq. 1, with the purchase cost (C_p_) depending on the characteristic size of each piece of equipment, such as volume or area and then converted to installed equipment cost (C_BM_) using the appropriate installation factor (F_BM_), following methodologies reported in the literature [39, 43, 88–90]. When no such equation was available, the cost was estimated using the six-tenths rule (Eq. 2) from existing costed equipment in literature or from graphs relating the cost to the characteristic size from Peters et al*.* [90] and checked against the online cost estimator [42]. In the end, all installed equipment costs were adjusted to the year 2019, using the Chemical Engineering Plant Cost Index (CEPCI) from the Chemical Engineering Magazine [91], accessed through the University of Bath Library, as per Eq. 3. The cost of manufacture was calculated using Eq. 4 [43]. All prices are expressed as USD ($) throughout. The cost of waste treatment was not considered in these scenarios:

1 $$C_{{{\text{BM}}}} = f\left( {F_{{{\text{BM}}}} , F_{{\text{d}}} , F_{{\text{p}}} , F_{{\text{m}}} } \right)C_{{\text{p}}} ,$$

where *C*_BM_ is the installed equipment cost, *F*_BM_ is the installation factor for the equipment, *F*_d_ is a correction factor for the type of equipment, *F*_p_ is the correction factor for the operation pressure, *F*_m_ is the correction factor for the material as the original equation is applicable to carbon steel only and *C*_p_ is the purchase equipment cost:

2 $$C_{{\text{p,b}}} = \left( {\frac{{C_{{\text{p,a}}} }}{{X_{{\text{a}}}^{{\text{n}}} }}} \right)X_{{\text{b}}}^{{\text{n}}} ,$$

where *X* is the characteristic size of the equipment a and b, respectively, and n is a superscript that takes values from 0.3 to 0.9 but usually takes the value of 0.6 if the exact superscript is not known (six-tenths rule). *C*_p,α_ and *C*_p_, b is the purchased equipment cost for equipment a and b, respectively,

3 $$C_{{\text{BM,b}}} = \frac{{{\text{CEPCI}}_{{\text{b}}} }}{{{\text{CEPCI}}_{{\text{a}}} }}C_{{\text{BM,a}}} ,$$

where *C*_BM,b_ is the installed equipment cost for the year b (unknown), CEPCI is the index for the year a and b, respectively, and *C*_BM,a_ is the known cost for year a (known):

4 $${\text{COM}} = 0.18\;{\text{FCI}} + 2.73\;C_{{{\text{OL}}}} + 1.23\;\left( {C_{{{\text{RM}}}} + C_{{{\text{UT}}}} + C_{{{\text{WT}}}} } \right),$$

where COM is the cost of manufacture, *C*_OL_ the labour cost, *C*_RM_ the raw materials cost, *C*_UT_ the utilities cost and *C*_WT_ the cost of waste treatment.

## Data Availability

The datasets supporting the conclusions of this article are all included within the article.

## Abbreviations

ALB: Airlift bioreactor
BCR: Bubble column bioreactor
CEPCI: Chemical Engineering Plant Cost Index
COM: Cost of manufacture
DCW: Dry cell weight
f.o.b.: ‘Free on-board’ cost
FCI: Fixed capital investment
LCA: Life cycle assessment
OUR: Oxygen uptake rate
PFD: Process flow diagram
SCO: Single-cell oils
STR: Stirred tank bioreactor
TEA: Techno-economic analysis
TRS: Total reducing sugars
USDA: United States Department of Agriculture
